# Outcomes in uncomplicated β-hemolytic Streptococcal bloodstream infections transitioned from IV to oral antimicrobial therapy

**DOI:** 10.1017/ash.2025.10109

**Published:** 2025-08-29

**Authors:** Mackenzie R. Keintz, Cristina Torres, Molly M. Miller, Trevor C. Van Schooneveld, Bryan T. Alexander, Elizabeth Lyden, Jihyun Ma, Jasmine R. Marcelin

**Affiliations:** 1 University of Nebraska Medical Center, Omaha, NE, USA; 2 Nebraska Medicine, Omaha, NE, USA

## Abstract

**Objective:**

To evaluate clinical outcomes in patients with uncomplicated β-hemolytic *Streptococcus* spp. bloodstream infections (BSI) transitioned to oral antimicrobial therapy (OAT) compared with those that remain on intravenous antimicrobial therapy.

**Design:**

Retrospective cohort study.

**Setting:**

Tertiary academic hospital.

**Methods:**

This retrospective cohort study included adult patients hospitalized between 1/1/2013 and 12/31/2019 diagnosed with uBSI due to β-hemolytic streptococci. Patients were excluded if BSI was due to endovascular, central nervous system, or bone/joint infection or patient was immunosuppressed or died within 72 hours of identification of BSI. We compared outcomes including: 30-day mortality, antimicrobial therapy, BSI relapse, 30-day rehospitalization, adverse drug events, and reversion to IV therapy. Fisher’s exact test was used for categorical variables; Mann – Whitney test and Independent T-test for continuous variables.

**Results:**

232 BSIs were included. OAT was used in 152 (65%). Cohort demographics were similar. Mortality was also similar between cohorts (2% vs 6% *P* = .13). Hospital length of stay was shorter in the OAT cohort with a median of 5 days (interquartile range 4.00, 8.00) versus 8 (5.00, 16.00) in the IV group (*P* < .0001). Patients transitioned to OAT were more likely to finish antibiotics outpatient (93% vs 62% *P* < .001).

**Conclusion:**

For β-hemolytic *Streptococcus* uBSI, OAT was associated with decreased length of stay without adverse clinical outcomes. Opportunities exist to modify clinical management of uBSI.

## Introduction

Bloodstream infections (BSI) are a common cause of morbidity and mortality in the United States with an incidence estimated between 113 and 204 per 100,000 persons per year. Incidence of beta-hemolytic streptococcal BSI has been estimated to be 14.4/100,000 persons per year in population-based studies.^1^ Although beta-hemolytic *Streptococcus* spp. compose only a small portion of BSI, their incidence has been increasing.^2–5^

Historically, BSI were treated with prolonged courses of intravenous (IV) antimicrobial therapy leading to longer durations of hospitalization, increased healthcare costs, and increased healthcare resource utilization through frequent need for outpatient parental antibiotic therapy (OPAT).^6–8^ In addition, administration of IV antibiotics typically requires a central venous catheter (CVC) with the associated risk of complications. Patients who are clinically improving with a controlled source of infection are often considered candidates for oral antimicrobial therapy (OAT). OAT has been demonstrated to be effective in gram-negative BSI.^9^ Beta-hemolytic streptococci are reliably susceptible to many oral agents with low MICs, making them ideal candidates for definitive oral antimicrobial therapy.^10^ Despite the availability of affordable and highly active oral agents, limited data regarding this approach are available for gram-positive organisms, including beta-hemolytic streptococci.^11–15^

We evaluated the outcome of oral antimicrobials as definitive therapy compared with a course of fully IV antimicrobial therapy in beta-hemolytic *Streptococcus* uncomplicated BSI (uBSI) at one large, Midwestern academic institution.

## Methods

This single-center retrospective cohort study included adult patients hospitalized between 1/1/2013 and 12/31/2019 diagnosed with uBSI secondary to beta-hemolytic *Streptococcus*. uBSI was defined as an event without persistent bacteremia or fever > 72 hours after initiation of active antimicrobial therapy and without metastatic site of infection. Patients were excluded if they had immunocompromising conditions which were defined as solid organ transplant on immunosuppression, hematopoietic bone marrow transplant within the last 12 months or on therapy for graft versus host disease, cancer on active chemotherapy, rheumatologic condition on biologic or chronic prednisone > 20 mg per day, neutropenia (ANC < 1000), or advanced HIV (CD4 < 200). BSI episodes were excluded if due to endovascular, central nervous system (CNS), or bone/joint infection without definitive surgical management (ie, amputation). Additionally, patients were excluded if they died within 72 hours of BSI onset as organism identification and susceptibility would not have been available. Multiple episodes of BSI per patient could be included if separated in time by > 90 days after therapy completion or if a different *Streptococcus* species was isolated. Polymicrobial BSIs were included if all other inclusion criteria were met. BSI with the same organism within 90 days of therapy completion was considered BSI relapse. Chart review was performed by two independent reviewers (MK and CT). The primary reviewer audited a sample of cases from the secondary reviewer to ensure concordance.

We compared patients treated exclusively with IV antibiotics to those transitioned from IV antibiotics to oral antibiotics (OAT cohort). The primary outcome was all-cause 30-day mortality from BSI onset. Secondary outcomes included infectious disease (ID) consultation, antimicrobial choice and duration, BSI relapse, 30-day rehospitalization, adverse drug events, reversion to IV therapy, and a composite outcome consisting of 30-day mortality, BSI relapse, and 30-day rehospitalization. Adverse effects evaluated included *C. difficile* infection, catheter-related BSI (CRBSI), line-related thrombotic event, rash, gastrointestinal effects, acute kidney injury, metabolic derangements, bone marrow suppression, line-associated erythema, line dislodgement, or other. OAT dosage was classified *a priori* as “standard”, “high dose”, or “non-standard” dosing schedules as defined by drug label and available data when possible, as well as expert opinion of ID pharmacist investigators (Supplemental Table 1). Antibiotic regimens were further classified as optimal (adequate dosing, bioavailable agent, adequate peak serum concentration, routinely efficacious vs beta-hemolytic *Streptococcus*) or suboptimal (inadequate dose, poorly bioavailable agent, inadequate peak serum concentration, intrinsic resistance of beta-hemolytic *Streptococcus*) (Table 1). Fisher’s exact test was used for categorical variables; Mann-Whitney test and independent t-test were used for continuous variables. A *P* value < .05 was considered statistically significant, and analysis was conducted using SAS 9.4.

**Table 1.** Oral antibiotic utilized and their bioavailability and susceptibilities

Antibiotic	Number of episodes utilized (N = 152)	Bioavailability	MIC90^10^	Percent susceptible^20,21^
**Optimal regimens**				
High dose amoxicillin^**^	23 (15%)	70 – 80%^19^	< .25	100%
Amoxicillin/clavulanate	27 (18%)	70 – 80%^19^	< .5	100%
High dose cephalexin^**^	13 (9%)	95%^19^	^*^	100%^*^
Cefadroxil	1 (1%)	90%^19^	*	100%^*^
Ciprofloxacin	1 (1%)	70%^19^	1	93%
Levofloxacin	22 (14%)	99%^19^	1	98%
Linezolid	4 (3%)	100%^23^	2	100%
Moxifloxacin	2 (1%)	90%^24^	.25	98%
Trimethoprim/sulfamethoxazole	5 (3%)	100%^19^	< .5	99%
**Suboptimal regimens**				
Standard dose amoxicillin^**^	18 (12%)	70 – 80%^19^	< .25	100%
Azithromycin	1 (1%)	40%^25^	> 4	68%
Cefdinir	5 (3%)	21 – 25%^19^	^*^	100%^*^
Cefuroxime	13 (9%)	30 – 60%^26^	^*^	100%^*^
Standard dose cephalexin^**^	8 (5%)	95%^19^	^*^	100%^*^
Clindamycin	12 (8%)	90%^27^	> 2	83%
Doxycycline	10 (7%)	95%^28^	> 8	47%
Metronidazole	2 (1%)	100%	N/A	N/A
Other	2 (1%)	N/A	N/A	N/A

* oral cephalosporin susceptibility inferred from penicillin G (MIC90 = < .06, % susceptible 100%) percent susceptible determined via CSLI breakpoints when available or EUCAST breakpoints.

** Dosing defined in supplement 1.

## Results

A total of 321 beta-hemolytic *Streptococcus* BSIs were screened, of which 89 were excluded due to death within 72 hours, immunosuppression, complicated BSI, outpatient management, or age < 19 years. OAT was used in 152 (65%) (Table 2). The most common source of bacteremia was skin and soft tissue infection in both cohorts. Group B *Streptococcus* was the most commonly isolated organism overall (*n* = 92/232, 40%), but Group A *Streptococcus* was the most common organism in the IV only cohort (*n* = 29/80, 36% *P* = .12). ID consultation was obtained in 55% of the OAT cohort and 67% of the IV only cohort (*P* = .09). The median PITT bacteremia score was 1 in the OAT cohort (interquartile range (IQR) 1.00, 2.00) versus 2 (IQR 1.00, 4.00) in the IV only cohort (*P* = .005).

**Table 2.** Demographics in uBSI secondary to beta-hemolytic *Streptococcus* spp in intravenous (IV) only vs IV to oral antimicrobial therapy (OAT)

	IV only (N = 80)	OAT (N = 152)	*p*-value
Gender^*^			
Female	27 (34%)	75 (49%)	
Male	53 (66%)	77 (51%)	.03
**Race/Ethnicity**			
American Indian/Alaska native	2 (3%)	3 (2%)	
Asian	0 (0%)	1 (1%)	
Black	11 (14%)	19 (13%)	
White	63 (80%)	121 (79%)	
Hispanic	4 (5%)	7 (5%)	
Unknown	0 (0%)	1 (1%)	.99
**Insurance**			
Private	14 (18%)	23 (15%)	
Medicare	43 (55%)	98 (65%)	
Medicaid	10 (13%)	17 (11%)	
Self-pay	11 (14%)	13 (9%)	.43
**Age (Years)**			
Median (IQR)	65 (49.0, 71.5)	61 (49.5, 75.5)	.15
**Pitt bacteremia score**			
Median (IQR)	2 (1.0, 4.0)	1(1.0, 2.0)	<.001
**Lancefield grouping**			
Group A Group B Group C/G Group F Other	29 (36%) 28 (35%) 20 (25%) 3 (4%) 0	33 (22%) 64 (42%) 48 (32%) 4 (3%) 3 (2%)	.12
**Source of infection** Urinary tract infection Pyelonephritis Pneumonia Skin and soft tissue infection Intraabdominal infection Line-related bacteremia Unknown Other	2 (3%) 0 (0%) 3 (4%) 44 (56%) 2 (3%) 6 (8%) 12 (15%) 10 (13%)	5 (3%) 2 (1%) 9 (6%) 97 (64%) 6 (4%) 1 (1%) 18 (12%) 13 (9%)	

* No patients identified as non-binary in this study.

Thirty-day mortality was similar between the two cohorts (2% vs 6% *P* = .13). Hospital length of stay was shorter in the OAT cohort with a median of 5 days (4.00, 8.00) versus 8 (5.00, 16.00) (*P* < .0001). Total duration of therapy similar between cohorts (14 (IQR 12.00, 16.00) vs 16 (14.00, 17.00) days *P* = .09). Patients in the OAT cohort received a median of 7 days of IV therapy (IQR 5.00, 10.00) prior to transition to oral antibiotics (Table 3). Patients transitioned to OAT were more likely to finish their antibiotic course outpatient (93% vs 62% *P* < .001). Adverse effects were more common in the IV only cohort (71% vs 86%, *P* = .01). There was no difference between cohorts for an individual category of adverse effect. Only half of the patients in the IV only cohort received a long-term catheter (73% PICC, 20% midline, 8% tunneled central line). There were no reported episodes of CRBSI. The composite outcome including 30-day mortality, 30-day rehospitalization, and recurrence of BSI was not different between cohorts (21% vs 30% *P* = .15).

**Table 3.** Outcomes in uBSI secondary to beta-hemolytic *Streptococcus* spp in intravenous (IV) only vs IV to oral antimicrobial therapy (OAT)

	IV only (N = 80)	OAT (N = 152)	*p*-value
**30-day mortality**			
	5 (6%)	3 (2%)	.13
**Recurrence of infection**			
	2 (3%)	8 (5%)	.50
**30-day rehospitalization**			
	19 (24%)	29 (19%)	.39
**Composite outcome**			
	24 (30%)	32 (21%)	.15
**Length of stay (days)**			
Median (IQR)	8 (5.0, 16.0)	5 (4.0, 8.0)	
**Duration of therapy (days)**			
Median (IQR)	16 (14.0, 17.0)	14 (12.0, 16.0)	.09
Time to OAT transition	–	7 (5.0, 10.0)	

The most used oral agent was amoxicillin (*n* = 41/152, 27%). Forty-five percent (*n* = 69/152) of OAT patients were on an antibiotic regimen that was suboptimal (Table 1). Reversion to IV therapy from OAT occurred in 11 patients (7%). An in-depth review revealed that 7/11 (64%) of the patients reverted to IV therapy due to worsening infection, of which 3/7 (43%) were secondary to inadequate source control that required further drainage. Of the 4 who were changed for non-infection-related reasons, two patients were not adherent to OAT, one was no longer tolerating oral medications, and one was restarted on IV antibiotics due to an unrelated change in clinical status. BSI relapse was rare, occurring in 5% (8/152) of patients in the OAT cohort compared to 3% (*n* = 2/80) in the IV-only cohort (*P* = .5). Review of patients with BSI relapse revealed 5/10 (50%) were secondary to poor source control requiring further intervention. Four patients had no identifiable reason for recurrence of BSI, and one patient was on a suboptimally dosed beta-lactam.

## Discussion

OAT was not associated with increased mortality in this single-center, retrospective review of beta-hemolytic Streptococcal bacteremia. Our findings agree with the increasing body of literature around this topic.^14–16^ Transition to OAT was associated with a shorter length of stay, possibly due to avoidance of challenges associated with planning outpatient intravenous therapy or less severe illness. This was highlighted by the increased likelihood of completing therapy outpatient on OAT versus IV only.

The duration of therapy exceeded the historical recommendation of 14 days of therapy in our study in patients continued on IV therapy. Limited retrospective data suggests that shorter duration may be effective.^16,17^ Infectious disease consultation was only pursued in about half of OAT patients and only about two-thirds of patients in the IV only cohort. Studies have demonstrated ID consultation leads to improved outcomes and more appropriate therapies.^18^

Despite evidence that OAT is an acceptable alternative to IV therapy, no guidelines exist yet to aid clinicians in this decision-making process. Although there were few episodes of mortality, recurrence of infection, and reversion to IV therapy, many of those rare cases were secondary to inadequate source control. The use of suboptimal antibiotic regimens such as doxycycline or underdosed beta-lactams in our study supports that further guidance on this topic would be helpful to clinicians. Forty-five percent of antibiotics utilized for OAT in our study were suboptimal agents (Table 1). Certain antibiotics are poor candidates for treating severe infections such as bloodstream infections because they are poorly absorbed, such as cefdinir and cefuroxime, which may lead to treatment failure.^19^ Some agents are absorbed well but have a low peak serum concentration and high volume of distribution which is not sufficient for treating bloodstream infections, such as tetracyclines and macrolides. Other agents may be inappropriate for beta-hemolytic *Streptococcus* due to increasing rates of acquired or intrinsic resistance, such as clindamycin, azithromycin, and metronidazole^10,20,21^ (Table 1). Preferred oral antibiotics for treating beta-hemolytic *Streptococcus* BSI include levofloxacin, trimethoprim-sulfamethoxazole, amoxicillin, amoxicillin/clavulanate, cephalexin, cefadroxil, and linezolid based on bioavailability, susceptibilities, and adequate peak serum concentrations^20,21^ (Table 1). Adequate dosing schedules should be utilized in BSI. Studies in gram-negative bacteremia have noted treatment failure when lower or infrequent dosing of beta-lactam antibiotics was used.^9^ This difference was not reflected in our study; however, our study had a relatively long IV lead in (average 8.3 days) and beta-hemolytic Streptococci have lower MICs to beta-lactam antibiotics than gram-negative organisms. Despite this, we would propose utilizing higher-dose beta-lactams and fluoroquinolones when transitioning to OAT in BSI particularly when early transition to OAT is utilized (supplement 1).

This study’s limitations stem from its retrospective design which could introduce potential selection bias including cohort selection and outcomes. Although all patients evaluated met criteria for uBSI when initial antimicrobial decisions were made, some patients were more acutely ill from their infection. The median PITT bacteremia score was higher in the IV-only cohort, suggesting more severe presentation of bacteremia, which may have influenced the decision not to transition to OAT for these patients. Despite presenting with more acute illness, many patients may have been candidates to switch to OAT once stabilized. Additionally, there was a greater prevalence of Group A *Streptococcus* in the IV cohort, which may have influenced this decision as well due to concern over toxin-mediated effects. In addition, a prolonged course of IV therapy was utilized prior to transition so outcomes may be reflective of an adequate course of therapy prior to transition to oral antimicrobial therapy which is supported by recent data that shorter courses may be effective for Streptococcal BSI.^17,22^

In conclusion, transition to OAT in patients with uBSI due to beta-hemolytic *Streptococcus* was not associated with increased mortality in this retrospective, single-center review. Transition to OAT resulted in shorter hospital length of stay without compromising clinical outcomes. In transition to OAT, it is important to optimize the dose and agent for beta-hemolytic BSI. Further prospective studies are needed to determine ideal candidates to transition to OAT.

## Supporting information

10.1017/ash.2025.10109.sm001 Keintz et al. supplementary material Keintz et al. supplementary material

## Supplementary material

The supplementary material for this article can be found at https://doi.org/10.1017/ash.2025.10109
