# Supplementary material for: Outcomes in uncomplicated β-hemolytic Streptococcal bloodstream infections transitioned from IV to oral antimicrobial therapy
Source: Antimicrob Steward Healthc Epidemiol. 2025 Aug 29;5(1):e194. doi: 10.1017/ash.2025.10109 (PMC12418230; doi:10.1017/ash.2025.10109)
Supplement: Keintz et al. supplementary material [file S2732494X25101095sup001.docx]

Supplemental 1: Dosing protocol

- Amoxicillin
  - Standard: 500mg q6 – 8 hr, 875mg q 12 hours
  - High Dose: 1g q 6-8 hours
- Amoxicillin/clavulanate
  - Standard: 875/125mg q12hr, 500/125mg q8hr
  - High Dose: 875/125mg q8h, 2000/125mg XR q12h
- Azithromycin
  - Standard: 500mg daily
  - High Dose: NA
- Cefadroxil
  - Standard: 500mg q12h
  - High Dose: 1g q12h
- Cefdinir
  - Standard: 300mg q12h
  - High Dose: NA
- Cefpodoxime
  - Standard: 100mg q12h, 200mg q12h
  - High Dose: 400mg q12h
- Cefuroxime
  - Standard: 250mg q12h
  - High Dose: 500mg q12h
- Cephalexin
  - Standard: 250mg q6h, 500mg q8-12h
  - High Dose: 500mg q6h, 1g q6-8h
- Ciprofloxacin
  - Standard: 250-500mg q12h
  - High Dose: 750mg q12h
- Clarithromycin
  - Standard: 500mg q12h
  - High Dose: NA
- Clindamycin
  - Standard: 150-450mg q6-8h
  - High Dose: 600mg q6-8h
- Doxycycline
  - Standard: 100mg q 12 hours
  - High Dose: NA
- Levofloxacin
  - Standard: 500q 24 hours
  - High Dose: 750mg q 24
- Linezolid
  - Standard: 600mg q12hr
  - High Dose: NA
- Moxifloxacin
  - Standard: 400mg q24h
  - High Dose: NA
- Minocycline
  - Standard: 100mg q12h
  - High Dose: NA
- Omadacycline
  - Standard: 300mg q24h
  - High Dose: NA
- Penicillin VK
  - Standard: 250-500mg q6-8h
  - High Dose: 1g q6-8h
- Rifampin
  - Standard: 300mg q12h, 600mg q24h
  - High Dose: 300mg q8h, 450mg q12h, 900mg q24h, 600mg q12h
- Tedizolid
  - Standard: 200mg q24h
  - High Dose: NA
- Trimethoprim-sulfamethoxazole
  - Standard: 1 DS q12h
  - High Dose: 1 DS q8h, 2 DS q8-12h
  - Non-standard: weight based
- Other
